# (A)voiding misdiagnosis: prediction of detrusor underactivity vs. bladder outlet obstruction using pre-urodynamic nomogram in male patients with LUTS

**DOI:** 10.1007/s11255-024-04093-7

**Published:** 2024-05-31

**Authors:** Karolina Garbas, Łukasz Zapała, Aleksander Ślusarczyk, Tomasz Piecha, Piotr Gwara, Aleksandra Żuk-Łapan, Hanna Piekarczyk, Piotr Zapała, Piotr Radziszewski

**Affiliations:** 1 Department of General, Oncological and Functional Urology, Medical University of Warsaw, Lindleya 4, 02-005 Warsaw, Poland; 2 Urodynamic Lab of Private Hospital “Prostalith”, Kielce, Poland

**Keywords:** Detrusor underactivity, Bladder outlet obstruction, Uroflowmetry, Lower urinary tract symptoms, Free flow curve shape

## Abstract

**Purpose:**

Our study aimed to develop a noninvasive model using a combination of the set of clinical data and uroflowmetry (UFL) to differentiate between detrusor underactivity (DU) and bladder outlet obstruction (BOO) in non-neurogenic male patients with lower urinary tract symptoms (LUTS).

**Methods:**

Data from 229 men with LUTS, diagnosed with DU or BOO on a pressure-flow study (PFS), were retrospectively analyzed, including medical history, Core Lower Urinary Tract Symptoms score (CLSS) questionnaire, UFL and PFS. Uni- and multivariate logistic regression were utilized for the prediction analyses.

**Results:**

Of the cohort, 128 (55.9%) patients were diagnosed with DU. A multivariate logistic regression analysis identified less prevalent nocturia (OR 0.27, *p* < 0.002), more prevalent intermittency (OR 2.33, *p* = 0.03), less prevalent weak stream (OR 0.14, *p* = 0.0004), lower straining points in CLSS (OR 0.67, *p* = 0.02), higher slow stream points in CLSS (OR 1.81, *p* = 0.002), higher incomplete emptying points in CLSS (OR 1.31, *p* < 0.02), lower PVR ratio (OR 0.20, *p* = 0.03), and present features of fluctuating (OR 2.00, *p* = 0.05), fluctuating-intermittent (OR 3.09, *p* < 0.006), and intermittent (OR 8.11, *p* = 0.076) UFL curve shapes as independent predictors of DU. The above prediction model demonstrated satisfactory accuracy (*c*-index of 0.783).

**Conclusion:**

Our 10-factor model provides a noninvasive approach to differentiate DU from BOO in male patients with non-neurogenic LUTS, offering a valuable alternative to invasive PFS.

**Supplementary Information:**

The online version contains supplementary material available at 10.1007/s11255-024-04093-7.

## Introduction

Detrusor underactivity (DU), although prevalent among patients with lower urinary tract symptoms, remains under-researched and poorly understood. Previous clinical studies indicate a presence of DU ranging from 9 to 28% in men under 50 years old, with even higher incidence rate as age advances [1]. According to the International Continence Society (ICS), DU is defined as “a contraction of reduced strength and/or duration, resulting in prolonged bladder emptying and/or failure to achieve complete bladder emptying within a normal time span” [2].

While a pressure-flow study (PFS) remains the gold standard for diagnosing DU, its cost, time-consuming nature, requirement for dedicated equipment, and need for highly trained urodynamics experts limit its accessibility. One cannot forget about the increased likelihood of infectious complications, especially in individuals with elevated PVR [3, 4]. Therefore, there is a need to develop a noninvasive, alternative approach for diagnosing DU, particularly useful, when PFS is unavailable or not accepted by a patient.

DU manifests as prolonged urination time, often accompanied by a sensation of incomplete emptying, hesitancy, reduced sensation of filling, and a weak stream [5]. These nonspecific symptoms pose challenges in differentiating DU from other causes of lower urinary tract symptoms (LUTS), notably bladder outlet obstruction (BOO). Accurate differentiation between those two voiding dysfunctions is crucial, since patients diagnosed with DU may not benefit from the proposed surgical treatment, as opposed to those with BOO [6]. Several studies have attempted to develop a noninvasive approach to diagnosing DU. Some researchers relied solely on medical history and identified symptoms such as decreased urinary stream, hesitancy or urgency as potential indicators of BOO, when differentiating from DU [7]. Others incorporated noninvasive UFL parameters, such as UFL curve shapes or PVR Ratio, along with symptoms and questionnaires to construct a prediction nomogram [8–10]. Nevertheless, definitive set of factors distinguishing DU from BOO remain elusive, which imposes the need for further development of noninvasive diagnostic method [1].

This is particularly crucial given the profound impact DU can have on both quality of life and healthcare costs. A study by Uren et al. revealed that symptoms such as high urinary frequency, nocturnal voids, and urgency led to over 27% of patients planning their daily activities around the availability of toilets. Additionally, almost 15% of patients experienced daytime somnolence due to disrupted sleep patterns. The resulting disturbances in social, work, or physical activities were reported as highly bothersome. Moreover, these symptoms affected self-image and confidence, leading to feelings of embarrassment in certain situations and impacting relationships with family and friends, including the sex lives of some patients [11]. Furthermore, according to a study by Sexton et al., patients with mixed storage, voiding, and post-micturition LUTS have an average of 5.9 visits to healthcare professionals per year, resulting in significant healthcare costs [12]. This underscores the issue of under-treatment or primary misdiagnosis among some patients.

The aim of our study was to identify noninvasive test parameters and symptoms specifically associated with DU, in contrary to BOO. Additionally, we sought to develop a model based on clinical data and uroflowmetry to differentiate impaired detrusor contractility from BOO in non-neurogenic male patients with LUTS.

## Materials and methods

### Study design and population

**Fig. 1:**
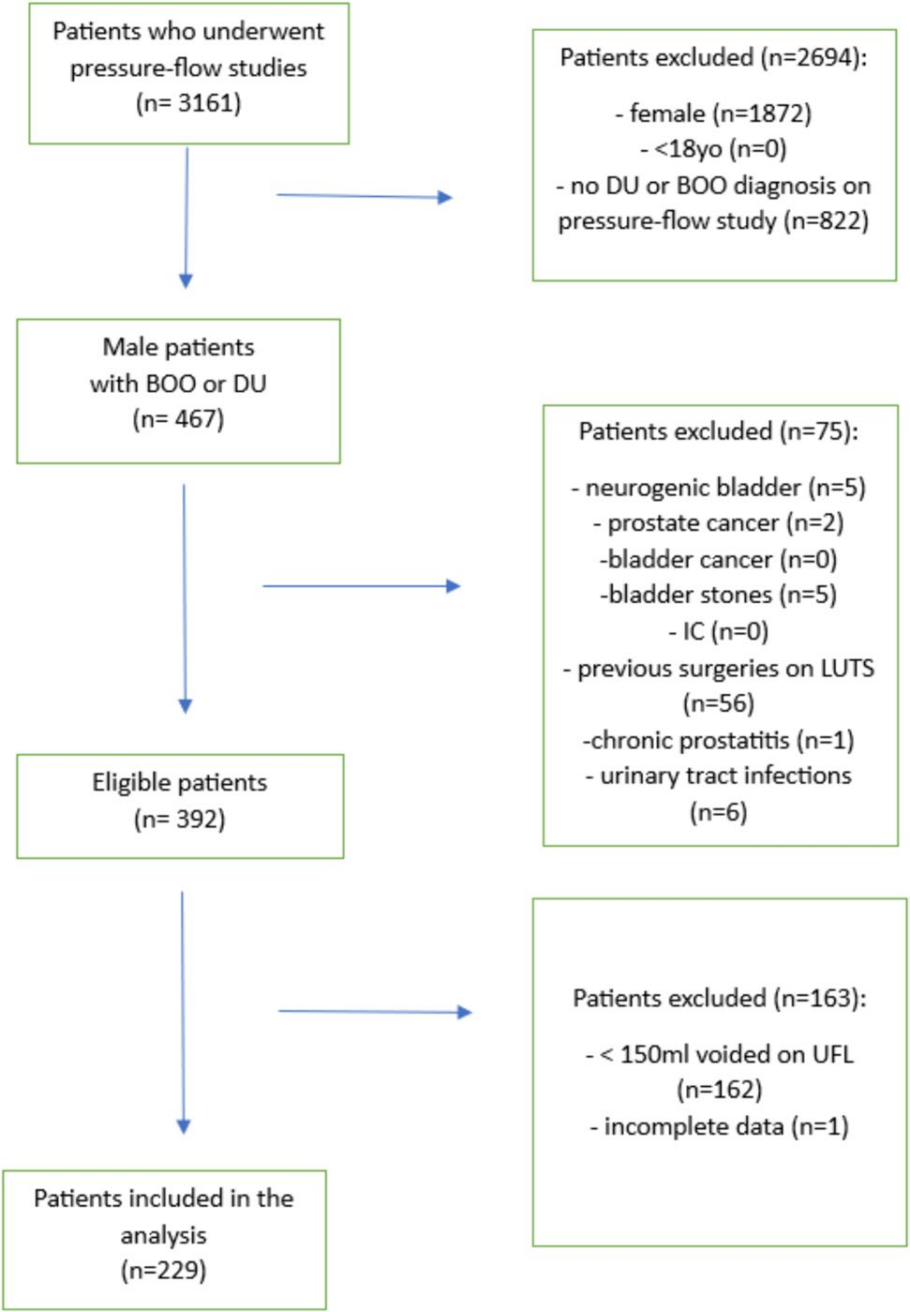
Flow chart of patients included in the study. *DU* detrusor underactivity, *BOO* bladder outlet obstruction, *UFL* uroflowmetry, *LUTS* lower urinary tract symptoms, *IC* interstitial cystitis

We retrospectively reviewed clinical data of consecutive 3161 patients who underwent pressure-flow studies between 2012 and 2022 at two outpatient clinics. The patients were referred for the urodynamic examination due to new-onset or persistent LUTS, failure of conservative treatment, planned urological surgery, failure of invasive approach or planned kidney transplant. The cohort was tested negative for urinary tract infection with urine culture performed prior to the PFS. Patients underwent evaluation by routine clinical tests, namely complete medical history including comorbidities and drug treatment, a Core Lower Urinary Tracts Symptoms score (CLSS) questionnaire [13], and a basic neurological exam [14]. Subsequently, a free flow study and a pressure-flow study were performed. The inclusion criteria were as follows: male gender, age > 18 years old, LUTS, final diagnosis of DU or BOO in the conducted pressure-flow study, uroflowmetry tests performed immediately prior to undergoing pressure-flow study, voided volume on uroflowmetry > 150 ml, informed consent. The exclusion criteria involved the following: neurogenic bladder (any history of neurologic disease), chronic prostatitis, interstitial cystitis, bladder cancer, prostate cancer, bladder stones, prior benign prostatic hyperplasia surgery, incomplete data. The final number of patients included in the study was 229 (Fig. 1). The study was approved by the local Bioethics committee of the Medical University of Warsaw (no. AKBE/335/2023) and was conducted in accordance with the ethical standards outlined in the Declaration of Helsinki.

### Urodynamic examination

Pressure-flow studies were performed by urodynamic-trained experts according to the International Continence Society (ICS) Good Urodynamics Practices protocol [15]. One used Medtronic devices (Duet Logic G/2, type: 9032A0173) to conduct free flow uroflowmetry with subsequent pressure flow studies with electromyography. The data were stored in the Medtronic software and analyzed according to the ICS recommendations [16].

Uroflowmetry parameters included maximum flow (*Q*_max_), mean flow (*Q*_mean_), voided volume, time to *Q*_max_, post-void residual urine (PVR) and curve shapes, classified as following possible shapes: bell-shaped, prolonged, fluctuating, intermittent, fluctuating-intermittent, plateau. In case of 47 patients, whose UFL curves had evident features of more than 1 of the aforementioned curve shapes, we assigned two shapes to describe their curves (plateau and fluctuating in 29 cases, plateau and fluctuating-intermittent in 18 cases). PVR was measured with the use of abdominal ultrasound after free uroflowmetry and PVR ratio (PVR-R) was calculated as the percentage of PVR to bladder volume (voided volume + PVR). Pressure-flow study was performed promptly after uroflowmetry. Detrusor underactivity was defined as a Bladder Contractility Index (BCI) less than 100, using the equation, BCI = Pdet*Q*_max_ + 5*Q*_max_ [17]. Bladder outflow obstruction (BOO) was defined as a BOO Index (BOOI) greater than 40 using the equation, BOOI = Pdet*Q*_max_ − 2*Q*_max_ [17]. Information from every pressure-flow study underwent meticulous screening for artifacts formation and was manually entered into the database. This approach ensured accuracy and helped to prevent errors that could arise from automated data extraction methods.

### Statistical analysis

Baseline patient characteristics were presented as medians with interquartile ranges for continuous variables and numbers with percentages for categorized variables. Differences in continuous variables were compared using the Mann–Whitney *U* test, while categorized variables were evaluated with Fisher’s exact test. Logistic regression was utilized for uni- and multivariate analyses. Multivariable analysis was performed with a stepwise selection of variables selected based on univariable analyses. Additional variables, such as age and PVR Ratio were considered due to their clinical importance and because their association with DU was proven in previous studies.

In the stepwise selection procedure, variables were added to the model based on a significance level of 0.2, and once included, they were reevaluated at each step and removed if their *p*-value exceeded 0.2, continuing until no further improvement was possible. Only statistically significant variables entered the final model. Odds ratios with 95% confidence intervals were derived via logistic regression. Sensitivity, specificity, positive predictive value (PPV) and negative predictive value (NPV) were also calculated for the model. A cutoff of 0.5 for model predicted probability was used to determine its diagnostic accuracy. Two-sided *p*-values < 0.05 denoted statistical significance. Statistical analysis was performed in the SAS software (version 9.4., SAS Institute Inc., Cary, NC, USA).

## Results

### Patients’ baseline characteristics

We enrolled 229 male patients, who presented with LUTS and were assessed with the use of the CLSS questionnaire, then underwent uroflowmetry and pressure-flow studies and had complete medical data available. Baseline characteristics of the patients are available in Table 1. Of the 229 patients, 128 (55.9%) were diagnosed with detrusor underactivity (DU), with a median BCI of 78.5 (IQR 57.8–88) and BOOI of 22 (7.5–40.6). In the DU group, 37 patients simultaneously exhibited features of bladder outlet obstruction, with corresponding BCI of 87.5 (IQR 78–93) and BOOI of 44.8 (IQR 42.8–52) (Online Resource 1). The control group comprised 101 patients (44.1%) diagnosed with BOO without DU, with a median BCI of 124.5 (IQR 112.5–141.5) and BOOI of 64.6 (IQR 52.6–80.8).

**Table 1:** Baseline characteristics of included patients with detrusor underactivity (DU) and with bladder outlet obstruction (BOO) without DU

Variables		BOO (*n* = 101)		DU (*n* = 128)		*p*-value
		No. of patients/median	% of patients/IQR	No. of patients/median	% of patients/IQR	
Age	Years	63	52–70	61.5	49–69.5	0.41
*Symptoms*						
Urgency		64	63.4	80	62.5	0.89
Frequency		60	59.4	85	66.4	0.28
Nocturia		87	86.1	94	73.4	0.02
Weak stream		80	79.2	87	68.0	0.06
Hesitancy		24	23.8	25	19.5	0.44
Intermittency		19	18.8	37	28.9	0.08
Straining		3	3.0	8	6.3	0.25
Incomplete emptying		36	35.6	56	43.8	0.21
Dribble		2	2.0	5	3.9	0.40
*CLSS questionnaire*						
Frequency	Points	2	0–2	2	0–2	0.19
Nocturia	Points	2	1–3	2	1–2	0.08
Urgency	Points	2	0–2	2	0–2	0.70
Slow stream	Points	2	1–3	3	1.5–3	0.09
Straining	Points	0	0–0	0	0–0	0.008
Incomplete emptying	Points	0	0–3	0	0–3	0.05
*Chronic diseases*						
DM		16	15.8	12	9.4	0.14
Hypothyroidism		5	5.0	6	4.7	0.93
*Drugs*						
Cholinolytics		6	6.1	17	13.8	0.06
Alpha-blockers		52	53.1	36	29.3	0.0003
Insulin		3	3.0	3	2.4	0.80
Oral drugs for DM		12	12.0	4	3.3	0.01
Statins		4	4.0	6	4.9	0.75

*CLSS* Core Lower Urinary Tract Symptoms score, *DM* diabetes mellitus

Patients diagnosed with detrusor underactivity had a median age of 61.5 years (IQR 49–69.5), while those diagnosed with bladder outlet obstruction had a median age of 63 years (IQR 52–70). Regarding comorbidities, within the DU group, diabetes mellitus (DM) was concurrent in 12 patients (9.38%) and hypothyroidism in 6 patients (4.69%). Whereas, in the BOO group, DM was present in 16 patients (15.8%) and hypothyroidism in 5 (5.0%). Moreover, prior to the first visit and urodynamic tests at our outpatient clinics, 17 patients (13.8%) in the DU group had been prescribed cholinolytics and 36 (29.3%) alpha-blockers by a general practitioner. In the BOO group, 6 patients (6.1%) were administered cholinolytics and 52 (53.1%) alpha-blockers. The continuation of baseline characteristics is available in Online Resource 2.

### Symptoms and CLSS questionnaire scores

The predominant lower urinary tract symptoms observed in patients with DU included nocturia in 94 cases (73.4%), weak stream in 87 (68.0%), and frequency in 85 (66.4%). It was followed by urgency, present in 80 patients (62.5%), incomplete emptying in 56 (43.8%) and intermittency in 37 (28.9%). The least frequently reported symptoms in DU patients were hesitancy in 25 (19.5%), straining in 8 (6.3%) and dribble in 5 patients (3.9%).

Meanwhile, among patients with BOO, nocturia was the most prominent symptom present in 87 cases (86.1%), followed by weak stream in 80 (79.2%), and urgency in 64 (63.4%). Sixty patients complained of frequency (59.4%), 36 of incomplete emptying (35.6%) and 24 of hesitancy (23.8%). The least common symptoms included intermittency in 19 cases (18.8%), straining in 3 (3%), and dribble in 2 (2%). CLSS questionnaire results are available in Table 1 and the continuation in Online Resource 2.

### Uroflowmetry and pressure-flow study

In terms of UFL, patients with DU exhibited a median Qmax of 12 ml/sec (IQR 8.8–14.6), a median voided volume of 284.5 (IQR 185–381.5) and a median PVR of 80 ml (IQR 30–200). Conversely, patients solely diagnosed with BOO demonstrated a median Qmax of 10.6 ml/sec (IQR 8.2–14.2), a median voided volume of 231 ml (IQR 167–344) and a median PVR of 100 ml (IQR 40–200). The predominant UFL curve shapes observed in DU patients were plateau in 47 cases (36.7%) and fluctuating in 45 (35.2%), mirroring the prevailing shapes in the BOO group (*n* = 51, 50.5%, *n* = 28, 27.7%, respectively).

Fluctuating-intermittent curve was present in 38 patients (29.7%), bell-shaped in 22 (17.2%), and intermittent in 5 (3.9%) patients with DU. Meanwhile, in BOO cohort, bell-shaped curve shape occurred in 22 (21.8%), fluctuating-intermittent in 18 (17.8%) and intermittent in only 1 (1.0%) patient. Prolonged curve shape was observed only in 4 patients, all of which were diagnosed with DU. PFS in the DU group revealed a median Pdet@*Q*_max_ of 37 cm H_2_O (IQR 20–51) and a median PVR of 178.5 ml (IQR 33.5–314.5), while in the BOO group, these parameters were 80 cm H_2_O (IQR 70–93) and 105 ml (IQR 30–176), respectively. Urodynamic findings are detailed in Table 2 and the continuation is available in Online Resource 2.

**Table 2:** Urodynamic findings of included patients with detrusor underactivity (DU) and with bladder outlet obstruction (BOO) without DU

		BOO (*n* = 101)		DU (*n* = 128)		*p*-value
		No. of patients/median	% of patients/IQR	No. of patients/median	% of patients/IQR	
*UFL parameters*						
Qmax	ml/sec	10.6	8.2–14.2	12	8.8–14.6	0.18
Voided Volume	ml	231	167–344	284.5	185–381.5	0.09
*Q*_mean_	ml/sec	5.5	4.1–7.6	5.7	4.1–7.9	0.64
*Q*_max_–*Q*_mean_ difference	ml/sec	4.9	3.5–7.1	5.7	4.1–7.8	0.06
Voiding time	sec	50	33–75	56.5	38–80	0.19
Time to *Q*_max_	sec	11	7–16	13	7–22	0.21
PVR	ml	100	40–200	80	30–200	0.46
PVR ratio		0.24	0.1–0.5	0.23	0.09–0.4	0.22
*UFL curve shapes*						
Bell-shaped		22	21.8	22	17.2	0.38
Prolonged		0	0	4	3.1	0.07
Fluctuating		28	27.7	45	35.2	0.23
Intermittent		1	1.0	5	3.9	0.17
Fluctuating-Intermittent		18	17.8	38	29.7	0.04
Plateau		51	50.5	47	36.7	0.04
*Pressure-flow study parameters*						
Qmax	ml/sec	8.1	6.6–11.1	6.6	4.5–8.8	< 0.0001
Pdet@*Q*_max_	cm H_2_O	80	70–93	37	20–51	< 0.0001
PVR	ml	105	30–176	178.5	33.5–314.5	0.001
BCI		124.5	112.5–141.5	78.5	57.8–88	< 0.0001
BOOI		64.6	52.6–80.8	22	7.5–40.6	< 0.0001

*UFL* uroflowmetry, *PVR* post-void residual urine, *Q*_*max*_ maximum flow, *Pdet@Q*_*max*_ detrusor pressure at maximum flow, *BCI* bladder contractility index, *BOOI* bladder outlet obstruction index

### Univariate logistic regression

Univariate logistic regression analysis showed that presence of nocturia (OR 0.45, 95% CI 0.22–0.89, *p* = 0.02), presence of weak stream (OR 0.56, 95% CI 0.30–1.02, *p* = 0.05), straining points in CLSS (OR 0.7, 95% CI 0.53–0.94, *p* < 0.02), incomplete emptying points in CLSS (OR 1.2, 95% CI 1.00–1.44, *p* < 0.05), fluctuating-intermittent curve shape in UFL (OR 1.95, 95% CI 1.03–3.67, *p* < 0.04), plateau curve shape in UFL (OR 0.57, 95% CI 0.34–0.97, *p* < 0.04), oral drugs for DM (OR 0.25, 95% CI 0.07–0.79, *p* < 0.02) were statistically significant factors for DU diagnosis on PFS. Complete univariate logistic regression analyses for the prediction of DU are demonstrated in Table 3.

**Table 3:** Univariate logistic regression analyses of factors predictive for DU in male patients with non-neurogenic LUTS with DU or BOO diagnosed on a PFS

Variables		Detrusor underactivity		
		OR	95% CI	*p*-value
Age		0.99	0.97–1.01	0.40
*Symptoms*				
Urgency		0.96	0.56–1.65	0.89
Frequency		1.35	0.79–2.32	< 0.28
Nocturia		0.45	0.22–0.89	**0.02**
Weak stream		0.56	0.30–1.02	**0.05**
Hesitancy		0.78	0.41–1.47	< 0.44
Intermittency		1.76	0.94–3.29	< 0.08
Straining		2.18	0.56–8.42	0.26
Incomplete emptying		1.40	0.82–2.40	0.21
Dribble		2.01	0.38–10.58	0.41
*CLSS questionnaire*				
Frequency	points	1.19	0.92–1.54	0.19
Nocturia	points	0.80	0.62–1.04	0.09
Urgency	points	1.03	0.80–1.32	0.83
Slow stream	points	1.16	0.94–1.43	< 0.16
Straining	points	0.70	0.53–0.94	**< 0.02**
Incomplete emptying	points	1.20	1.00–1.44	**< 0.05**
Overall CLSS score	points	1.04	0.96–1.12	0.37
*UFL*				
*Q*_max_	ml/sec	1.03	0.98–1.08	0.28
Voided volume	ml	1.00	0.99–1.00	0.25
*Q*_mean_	ml/sec	0.99	0.91–1.09	0.99
*Q*_max_–*Q*_mean_ difference	ml/sec	1.09	0.99–1.19	< 0.06
Voiding time	sec	1.004	0.99–1.01	< 0.39
Time to *Q*_max_	sec	1.007	0.99–1.02	0.24
PVR	ml	1	0.99–1.001	0.75
PVR ratio		0.49	0.14–1.72	0.26
*UFL curve shapes*				
Bell-shaped		0.75	0.39–1.44	0.38
Prolonged		–	–	0.98
Fluctuating		1.41	0.80–2.49	0.23
Intermittent		4.06	0.47–35.35	0.20
Fluctuating-Intermittent		1.95	1.03–3.67	**< 0.04**
Plateau		0.57	0.34–0.97	**< 0.04**
*Chronic diseases*				
Diabetes mellitus		0.55	0.25–1.22	0.14
Hypothyroidism		0.94	0.28–3.19	0.93
*Drugs*				
Insulin		0.81	0.16–4.09	0.80
Oral drugs for DM		0.25	0.07–0.79	**< 0.02**

*CLSS* Core Lower Urinary Tract Symptoms Score, *UFL* uroflowmetry, *PVR* post-void residual urine, *Q*_*max*_ maximum flow, *Q*_*mean*_ mean flow, *DM* diabetes mellitus

### Multivariate logistic regression

The final multivariate model incorporated 10 factors, including symptoms, CLSS questionnaire, UFL parameters and UFL curve shapes (Table 4). The model revealed that less prevalent nocturia (OR 0.27, 95% CI 0.12–0.61, *p* < 0.002), more prevalent intermittency (OR 2.33, 95% CI 1.08–5.00, *p* = 0.03), less prevalent weak stream (OR 0.14, 95% CI 0.05–0.42, *p* = 0.0004), lower score in CLSS straining points (OR 0.67, 95% CI 0.48–0.94, *p* = 0.02), higher score in CLSS slow stream points (OR 1.81, 95% CI 1.24–2.63, *p* = 0.002), higher score in CLSS incomplete emptying points (OR 1.31, 95% CI 1.05–1.63, *p* < 0.02), lower PVR Ratio (OR 0.20, 95% CI 0.05–0.87, *p* = 0.03), and presence of fluctuating curve shape (OR 2.00, 95% CI 0.99–4.05, *p* = 0.05), fluctuating-intermittent curve shape (OR 3.09, 95% CI 1.39–6.86, *p* < 0.006), intermittent curve shape (OR 8.11, 95% CI 0.80–82.50, *p* = 0.076) were significant predictors for DU diagnosis. The risk model constructed on the aforementioned factors for predicting DU diagnosis in patients with LUTS in the course of DU or BOO, achieved a satisfactory Harrell's concordance index (C-index) of 0.783. The sensitivity, specificity, PPV, and NPV of these clinical diagnostic criteria for urodynamic DU were 75.8%, 62.4%, 71.9%, and 67.0%, respectively.

**Table 4:** A multivariate logistic regression analysis for predicting DU in male patients with non-neurogenic LUTS

Variables		Detrusor underactivity		
		OR	95% CI	*p*-value
*Symptoms*				
Nocturia	0	Ref	–	
	1	0.27	0.12–0.61	**< 0.002**
Intermittency	0	Ref	–	
	1	2.33	1.08–5.00	**0.03**
Weak stream	0	Ref	–	
	1	0.14	0.05–0.42	**0.0004**
*CLSS questionnaire*				
Straining points		0.67	0.48–0.94	**0.02**
Slow stream points		1.81	1.24–2.63	**0.002**
Incomplete emptying points		1.31	1.05–1.63	**< 0.02**
*UFL parameters*				
PVR ratio		0.20	0.05–0.87	**0.03**
*UFL curve shapes*				
Fluctuating	0	Ref	–	
	1	2.00	0.99–4.05	**0.05**
Fluctuating intermittent	0	Ref	–	
	1	3.09	1.39–6.86	**< 0.006**
Intermittent	0	Ref	–	
	1	8.11	0.80–82.50	0.076

*CLSS* Core Lower Urinary Tract Symptoms Score, *UFL* uroflowmetry, *PVR* post-void residual urine

## Discussion

In the current study, we have proposed a novel approach for noninvasive testing to be utilized clinically for the prediction of DU in males with non-neurogenic LUTS, when differentiating from BOO. Our final model of good accuracy (*c*-index nearly 0.8) incorporated 10, both subjective and objective, factors of the following categories: free-flow curve shapes, uroflowmetry parameters, symptoms and symptoms’ severity assessed with the use of the CLSS questionnaire. These factors comprised fluctuating, fluctuating-intermittent and intermittent UFL curve shapes, lower PVR Ratio, less prevalent nocturia, more prevalent intermittency, less prevalent weak stream, lower straining points in CLSS, higher slow stream points in CLSS and higher incomplete emptying points in CLSS.

A particularly innovative aspect of our model is the incorporation of free-flow curve shapes, a parameter that has been explored in only few studies [9, 18]. We found that fluctuating curve shape, fluctuating-intermittent curve shape, and intermittent curve shape on the free-flow are predictive of DU, and not BOO. A study by Matsukawa et al. on males with non-neurogenic LUTS demonstrated results consistent with ours and showed that sawtooth and interrupted waveform curve shape on uroflowmetry are significant predictors of detrusor underactivity, distinguishing it from BOO [19]. Another study revealed that the prolonged/tailed shaped free urine flow curve patterns were independent predictors of BOO when differentiating from DU in female patients with voiding difficulty [18]. However, in our study only 4 patients with DU presented with prolonged curve shapes in UFL, rendering it non-diagnostic. Kocadag et al. compared flow rate curve patterns in female patients with LUTS and found that women without a prolonged void and bell-shaped traces had normal voiding urodynamics in 76% of cases, and the majority could be managed noninvasively. Patients with fluctuating and intermittent flow rate curves demonstrated a variety of urodynamic diagnoses, with a third of cases showing obstruction and a third showing detrusor underactivity. Plateau flow rate curve patterns were associated with urethral obstruction [9]. The aforementioned results underlined the significance of curve shapes in diagnosing the causes of LUTS in patients.

Another factor distinguishing DU from BOO in our model was the lower PVR Ratio (PVR-R). Rubilotta et al. introduced a novel functional parameter, the PVR Ratio, representing the ratio of post-void residual (PVR) urine to bladder volume (BV). Their study, using the ICS nomograms and BCI, revealed that median PVR-R and PVR were notably higher in obstructed and underactive males, compared to patients suffering from LUTS without BOO or DU [20]. Moreover, another study demonstrated that higher PVR-R was a significant predictor for BOO. However, the study compared patients with BOO to those without it, rather than specifically comparing DU to BOO [21]. Previous researches have investigated sheer PVR as a potential predictor of DU, however the results remain inconsistent. Our study did not identify PVR as a predictor of DU. Namitome et al. reported similar findings and demonstrated no significant difference in PVR and bladder voiding efficiency between patients with or without underactive detrusor [8]. Conversely, Jeong et al. observed a significantly increased PVR in male patients over 65 years old with LUTS and detrusor underactivity, compared to those with detrusor overactivity and bladder outlet obstruction [22].

Further variables included in our model encompassed the symptoms. Among these, we identified predictors of DU, as opposed to BOO, which included more prevalent intermittency, less prevalent weak urinary stream, less prevalent nocturia, lower straining score, higher slow stream score and higher incomplete emptying score on the CLSS questionnaire. In line with our findings, Gammie et al. observed a significantly higher prevalence of incomplete emptying and an interrupted urinary stream among patients with DU [7], aligning with our identification of intermittency and higher incomplete emptying score as predictors of DU in the current paper. Other research conducted by Çetinel et al. revealed that the presence of weak urinary stream was a significant predictor of BOO compared to DU and unclassified pressure-flow studies in females [18], a finding consistent with our observations in male population. However, the occurrence of weak urinary stream was also identified as a predictive factor for DU [8], when compared to all patients with non-neurogenic LUTS, such as overactive bladder, rather than solely when compared to those with BOO. Our analysis revealed that absence of weak stream in a patient is predictive of DU. However, if a patient already complains of weak stream, a higher slow stream score becomes a predictor of DU, indicating that if the weak stream occurs, it is more frequent and persistent compared to patients with BOO. A lower straining score was also indicative of DU in our study, suggesting that patients with DU tend to experience less straining, compared to those with BOO. This could be attributed to the nature of BOO, often involving an active obstruction below the bladder, which may require more additional abdominal pressure to overcome it. Additionally, the severity of the obstruction may fluctuate daily, which is potentially influenced by factors such as coexisting inflammation in the lower urinary tract, dietary habits, environmental factors, levels of physical activity and emotional stress or anxiety. However, research on this topic is currently very limited [23].

It is possible that the severity of lower urinary tract symptoms, rather than their sheer occurrence, plays an important role in distinguishing between DU and BOO. In this context, several studies on male population used mostly IPSS questionnaire [8, 24, 25]. However, we opted for the Core Lower Urinary Tract Symptoms score (CLSS) questionnaire proposed by Homma et al., as it is suitable for new patients, including those with multiple diseases, and without a definite diagnosis at first [13].

Our analysis identified less prevalent nocturia as a predictor of DU, a finding not confirmed by other studies distinguishing between DU and BOO [8, 25]. Nevertheless, this result appears plausible, given that the underactive bladder development may stem from damage to the bladder afferent and efferent pathways, or to the lumbosacral spinal cord [26]. Currently, significant emphasis is placed on neural mechanisms controlling the voiding process, particularly the afferent pathways responsible for monitoring bladder filling volume. The integrity of these pathways is crucial for the detrusor contraction efficiency. Therefore, any disruption to these nerves can lead to diminished bladder volume sensitivity, reduced detrusor contraction strength, or early termination of the voiding reflex. This can further impair voiding efficiency and result in hyposensitive bladder, leading to a lack of need to void at night [27], in contrast to an obstructed bladder outlet. This phenomenon may also account for disrupted bladder sensitivity during the day, and therefore higher incomplete emptying score that was also predictive of DU in our analysis.

In terms of urgency, previous studies indicated that less prevalent urgency was a predictive factor for DU when distinguishing it from BOO [8, 25], although this observation was not statistically significant in our study. The older age of patients included in these studies (median age = 70, IQR 65–76) and thus possibly more advanced damage to afferent and efferent bladder pathways, might potentially account for the discrepancy. Additionally, the study by Namitome et al. included patients with detrusor overactivity, present in 55% of cases [8], which could further explain the difference.

In our study, post-micturition dribble (PMD) was observed infrequently, with only 5 (3.9%) patients exhibiting this symptom in the DU group and 2 (2.0%) patients in the BOO group, rendering it non-diagnostic in our analysis. However, a study by Liu et al. demonstrated a correlation between PMD and voiding symptoms, along with an association with decreased *Q*_max_. This suggests a need for further investigation, particularly among older men, as the study noted a significant increase in PMD prevalence with age [28].

It is important to acknowledge that symptoms constitute a subjective factor in the diagnostic process, lean solely on physician–patient dialogue, and demand comprehensive explanation of the medical terms followed by a meticulously taken medical history. This might explain why some studies have indicated that isolated symptoms classified by the International Prostate Symptom Score (IPSS) could not effectively differentiate patients with DU from those with BOO [10, 22], contributing to discrepancies between research findings in this regard.

Finally, in our study, age was not identified as a significant predictive factor for detrusor underactivity, contrary to other researches. These studies indicated an increasing prevalence of DU with advancing age [8, 22]. However, it is important to note that in these studies, the median age of patients was notably higher, with one study reporting a median age of 70 (IQR 65–76) [8] and the other study revealing that 63,8% of men were over 70 years old [22]. In contrast, the median age of patients in our study with DU is 61.5 (IQR 49–69.5), indicating a relatively younger population. This age distribution may contribute to the lack of statistically significant association between older age and DU diagnosis in our study. Moreover, the incidence of age-related comorbidities was low in our cohort. Statins were taken by only 4 (4.0%) patients in the BOO group and 6 (4.9%) patients in the DU group (Table 1). While age-related comorbidities associated with ischemia and oxidative stress, such as arteriosclerosis, are known to decrease detrusor contractility [27], they are not pertinent to our relatively youthful and healthy study population.

The calculated values for sensitivity, specificity, PPV, and NPV for our multivariate model were 75.8%, 62.4%, 71.9%, and 67.0%, respectively. These values are comparable to or slightly better than other DU predictors and diagnostic methods. Recently, Matsukawa et al. compared intravesical prostatic protrusion (IPP) between patients with detrusor underactivity (DU) and those with bladder outlet obstruction (BOO). They found that a lower IPP was a significant predictor of DU, with an optimal cut-off value of 8.2 mm yielding a sensitivity of 77% and a specificity of 73% [19]. Additionally, Matsukawa et al. identified bladder voiding efficiency (BVE) as a clinical predictor of DU, with a cut-off value of 70% providing a sensitivity of 73% and a specificity of 57% [19]. One study demonstrated that patients with DU had higher PVR compared to the control group, with a cut-off of 147 mL yielding a sensitivity of 60.16% and a specificity of 72.97% for diagnosing DU [24]. A novel study by Ishikawa et al. proposed serum adiponectin level as a DU predictor, with a cut-off value of 7.9 μg/mL providing a sensitivity of 79% and a specificity of 90% [29]. Although promising, serum adiponectin level testing is currently not widely available and remains costly.

Despite variations among studies concerning DU, we are confident that our newly developed model can be integrated into routine clinical practice, particularly during the initial ambulatory visit of patients. This is especially relevant in cases where non-adherence or potential lack of follow-up is suspected, or if a patient expresses reluctance towards undergoing an invasive pressure-flow study. Lastly, there is a high-risk group of patients, particularly those prone to infectious complications, who would greatly benefit if practitioners refrained from invasive tests during the primary diagnostics of LUTS. Conducting a comprehensive medical interview, coupled with the swift and easily applicable Core Lower Urinary Tract Symptoms score (CLSS) questionnaire, followed by a detailed analysis of the UFL curve and PVR volume, proves to be both time-efficient and cost-effective. This approach enables prompt initiation of appropriate medication, facilitating timely management of DU or BOO.

### Limitations

There are several limitations to our study that should be acknowledged. Firstly, this was a retrospective analysis, which may introduce biases and limitations inherent to its study design. Secondly, we excluded patients who could not void more than 150 ml on UFL, including those with urinary retention, which eliminated patients with severe voiding conditions and might have affected our results. Additionally, the patient cohort included in our study consisted of individuals with non-neurogenic LUTS suggestive of both BOO and DU, which does not represent the general population, as it was a single geographic and ethnic population study, performed on white Polish males. Therefore, the results obtained in our study are not applicable as a screening tool for a broader population or females. Furthermore, we did not evaluate prostate volume in our study participants, which could have provided additional insights into the relationship between prostate size and voiding dysfunction. Finally, some patients included in the study had already commenced medication affecting voiding patterns before undergoing noninvasive and invasive urodynamic testing.

Despite these limitations, we believe that our study provides valuable insight, particularly given the scarcity of research differentiating DU from BOO based on symptoms and noninvasive test parameters, without reliance on PFS outcomes. The findings of our study offer potential means to distinguish between DU and BOO in males using clinical examination and analysis of UFL parameters, especially in situations where PFS is unavailable or not accepted by patients. This insight seems most valuable when determining the appropriate treatment, whether noninvasive or invasive, such as transurethral prostate resection, in patients exhibiting symptoms indicative of BOO and DU. External validation of our model is warranted, considering other geographic and ethnic populations, to further explore its applicability and compare it with other existing models and diagnostic tools.

## Conclusions

Ten factors were identified as predictors of detrusor underactivity in our study, which was done using a comprehensive database of non-neurogenic male patients who underwent pressure-flow studies. These factors include less prevalent weak urinary stream, less prevalent nocturia, more prevalent intermittency, lower straining score, higher slow stream score, and higher incomplete emptying score on the CLSS questionnaire, as well as lower PVR Ratio, fluctuating curve shape, fluctuating-intermittent curve shape, and intermittent curve shape on free-flow uroflowmetry. Utilizing these factors, we developed a predictive model for detrusor underactivity, aiming to estimate the likelihood of detrusor underactivity in clinical settings without the need for an invasive pressure-flow study.

## Supplementary Information

Below is the link to the electronic supplementary material.

Supplementary file1 (PDF 136 KB)

Supplementary file2 (PDF 105 KB)

## Data Availability

The data that support the findings of this study are available on reasonable request from the corresponding author.
