# Supplementary material for: (A)voiding misdiagnosis: prediction of detrusor underactivity vs. bladder outlet obstruction using pre-urodynamic nomogram in male patients with LUTS
Source: Int Urol Nephrol. 2024 May 31;56(11):3485–94. doi: 10.1007/s11255-024-04093-7 (PMC11464610; doi:10.1007/s11255-024-04093-7)
Supplement: Supplementary file 1 — Supplementary file1 (PDF 136 KB) [file 11255_2024_4093_MOESM1_ESM.pdf]

Online Resource 1. Baseline characteristics and urodynamic findings of DU+BOO subgroup within DU patients, compared to patients with either DU or BOO.

			DU+BOO (n=37)		Either DU or BOO (n=192)		
			No. of pts/ median	% of patients/ IQR	No. of pts/ median	% of patients/ IQR	P-value
Age		Years	64	56-69	62	49.5-70	0.47
Symptoms	Urgency		29	78.4	115	59.9	0.03
	Frequency		29	78.4	116	60.4	0.03
	Nocturia		32	86.5	149	77.6	0.22
	Weak stream		26	70.3	141	73.4	0.69
	Hesitancy		46	24	3	8.1	0.03
	Intermittency		10	27	46	24	0.69
	Straining		1	2.7	10	5.2	0.51
	Incomplete emptying		20	54.1	72	37.5	0.06
	Dribble		0	0	7	3.7	0.23
CLSS questionnaire	Frequency	points	2	1-2	2	0-2	0.18
	Nocturia	points	2	2-2	2	1-3	0.26
	Urgency	points	2	2-2	2	0-2	<0.01
	Slow stream	points	3	1-3	3	1-3	0.81
	Straining	points	0	0-0	0	0-0	0.80
	Incomplete emptying	points	3	0-3	0	0-3	0.03
UFL parameters	Qmax	ml/sec	10.6	8.3-14.2	11.1	8.5-14.7	0.45
	Voided Volume	ml	300	204-379	252.5	171.0-358.5	0.19
	Qmean	ml/sec	5.1	3.9-7.1	5.75	4.2-8.1	0.22
	Qmax-Qmean difference	ml/sec	5.9	3.9-7.7	5.4	3.8-7.5	0.86
	Voiding time	sec	62	48-85	53	33-74	0.02
	Time to Qmax	sec	11	7-15	12	7-21	0.28
	PVR	ml	80	30-200	97	40-200	0.85
	PVR Ratio		0.3	0.1-0.4	0.226	0.1-0.5	0.94
Pressure-flow study parameters	Bladder capacity	ml	367	315-430	367.5	302-455.5	0.86
	Compliance	ml/cm H2O	50.6	34.5-71.3	55.4	29.5-95.9	0.71
	Qmax	ml/sec	5.5	4.0-6.1	7.7	6.1-10.4	<0.0001
	Qmean	ml/sec	2.3	1.9-2.9	3.9	2.6-5.2	<0.0001
	Pdetmax	cm H2O	62	58-69	71.5	39.5-102.5	0.57
	Pdet@Qmax	cm H2O	60	53-60	61	29.5-80	0.98
	Voiding time	sec	71	59-91	66	45-87	0.23
	Voided volume	ml	151	99-238	244.5	151.5-314.5	0.003
	PVR	ml	244	73-303	116	24-247.5	0.02
	BCI		87.5	78-93	101	73.8-125.8	<0.01
	BOOI		44.8	42.8-52	41.4	16.9-65.5	0.15
UFL curve shapes	Bell-shaped		7	18.9	37	19.3	0.96
	Prolonged		1	2.7	3	1.6	0.62
	Fluctuating		11	29.7	62	32.3	0.76
	Intermittent		0	0	6	3.1	0.28

	Fluctuating-Intermittent		12	32.4	44	22.9	0.22
	Plateau		16	43.2	82	42.7	0.95
Chronic diseases	Diabetes mellitus		6	16.2	22	11.5	0.42
	Hypothyroidism		2	5.4	9	4.7	0.85
Drugs	Cholinolytics		6	16.2	17	9.2	0.20
	Alpha-blockers		13	35.1	5	40.8	0.52

CLSS- Core Lower Urinary Tract Symptoms, UFL- uroflowmetry, Qmax- maximum flow, Qmean- mean flow, PVR- post-void residual urine, Pdetmax- maximum detrusor pressure, Pdet@Qmax- detrusor pressure at maximum flow, BCI- bladder contractility index, BOOI- bladder outlet obstruction index
