# Supplementary material for: (A)voiding misdiagnosis: prediction of detrusor underactivity vs. bladder outlet obstruction using pre-urodynamic nomogram in male patients with LUTS
Source: Int Urol Nephrol. 2024 May 31;56(11):3485–94. doi: 10.1007/s11255-024-04093-7 (PMC11464610; doi:10.1007/s11255-024-04093-7)
Supplement: Supplementary file 2 — Supplementary file2 (PDF 105 KB) [file 11255_2024_4093_MOESM2_ESM.pdf]

Online Resource 2. Baseline characteristics of included patients with detrusor underactivity (DU) and with bladder outlet obstruction (BOO) without DU, continuation.

			BOO (n=101)		DU (n=128)		
			No. of pts/ median	% of patients/ IQR	No. of pts/ median	% of patients/ IQR	P-value
CLSS questionnaire	Urge incontinence	points	0	0-0	0	0-0	0.51
	Stress incontinence	points	0	0-0	0	0-0	0.12
	Pain in the bladder	points	0	0-0	0	0-0	0.18
	Pain in the urethra	points	0	0-0	0	0-0	0.07
	Overall CLSS score	points	8	6-10	8	6-11	0.36
Chronic diseases	Hashimoto		1	1	1	0.8	0.87
	Recurring UTIs		1	1	4	3.1	0.27
Drugs	Levothyroxine		3	3	3	2.4	0.80
	Neuroleptics		2	2	2	1.6	0.83
	Mirabegron		2	2	2	1.6	0.81
	Tricyclic antidepressant		0	0	1	0.8	0.36
	Baclofen		0	0	2	1.6	0.20
Pressure-flow study parameters	Bladder capacity	ml	350	289-435	385	310-460	0.14
	Compliance	ml/cm H2O	47.7	19.7-78.4	57	36-149.5	0.002
	Voiding time	sec	67	48-87	66.5	43-88.5	0.84
	Voided volume	ml	252	186-311	200.5	92-298	0.003
	Pdetmax	cm H2O	101	84-123	46.5	29-58.5	<0.0001
	Qmean	ml/sec	4.1	3.4-5.2	3	2.1-4.4	<0.0001

CLSS- Core Lower Urinary Tract Symptoms, Qmean- mean flow, Pdetmax- maximum detrusor pressure, UTI- urinary tract infection
